# Characteristics of the electron beam outside the applicator in an Elekta Versa HD Linac

**DOI:** 10.1007/s13246-025-01530-4

**Published:** 2025-03-18

**Authors:** Kapil Dev Maharaj, Simon Goodall, Mahsheed Sabet, Joshua Dass, Mounir Ibrahim, Talat Mahmood, Pejman Rowshanfarzad

**Affiliations:** 1 School of Physics, Mathematics and Computing, The University of Western Australia, 35 Stirling Highway, Mailbag M013, Crawley, WA 6009 Australia; 2 GenesisCare, Wembley, WA Australia; 3 Department Radiation Oncology, Sir Charles Gairdner Hospital, Nedlands, WA Australia; 4 Centre for Advanced Technologies in Cancer Research (CATCR), Perth, WA Australia

**Keywords:** Peripheral dose, Electron therapy, Applicator, Elekta versa HD, Linac, Out-of-field

## Abstract

Radiotherapy is an essential component of cancer treatment, but healthy tissues can be exposed to out-of-field doses, potentially causing adverse effects and secondary cancers. This study investigates peripheral doses outside the electron beam applicator in an Elekta Versa HD linear accelerator. Peripheral doses outside an electron applicator were measured using 6, 9, and 12 MeV beams at their respective maximum dose depths while maintaining a 100 cm source-to-surface distance. Measurements employed EBT3 films within Plastic Water DT phantoms. The influence of field size on penumbra width and peripheral doses were examined using various cutouts (6 × 6 cm², 10 × 10 cm², and a 5 cm diameter circle) within a 10 × 10 cm² applicator, with gantry and collimator angles set to 0 degrees. Additionally, the impact of collimator angles on penumbra width and peripheral doses was explored, enhancing the understanding of dose distribution. Measured profiles were also compared with those calculated using Monaco treatment planning system. Findings showed that both penumbra width and peripheral dose values increased with energy across different field sizes and collimator angles. Root Mean Square Deviation (RMSD) analysis indicated deviations of 1.8 mm for penumbra and 1.1% for peripheral doses between measured profiles and Treatment Planning System (TPS) predictions for all field sizes. Peripheral doses remained below 5% of the maximum dose at distances ranging from 10 to 15 mm away from the field edges, indicating acceptable tolerance levels (ICRU report 24). However, further dose reduction may be possible with additional shielding to keep doses as low as reasonably achievable. This study highlights the critical importance of considering peripheral doses in radiotherapy, emphasizing the need to evaluate the impact on healthy tissues outside the primary treatment area to ensure patient safety and mitigate long-term treatment-related side effects. The findings underscore the necessity of implementing appropriate measures to minimize peripheral doses.

## Introduction

The introduction of modern radiotherapy technologies has resulted in substantial improvement of the long-term survival rates for cancer patients. However, individuals who have successfully battled cancer still face an increased risk of developing subsequent malignancies. Current statistics indicate that approximately 17–19% of these survivors eventually experience a second form of cancer [1]. This phenomenon can be attributed to a combination of factors, including ongoing lifestyle choices, genetic predisposition, and prior treatments such as radiotherapy and chemotherapy. While radiotherapy accounts for about 5% of these treatment-related secondary malignancies, it is challenging to isolate its specific impact due to the numerous contributing factors at play [1]. According to data from the U.S. National Cancer Institute’s Surveillance, Epidemiology and End Results (SEER) program, the incidence of second malignancies has doubled from 1975 to 2009 [1]. Consequently, given that stochastic effects can induce cancer without a minimum dose threshold, it is imperative not to underestimate the significance of peripheral radiation dose and penumbra width in radiation therapy, particularly when it comes to the proximity of critical organs, such as the eyes. According to the International Committee for Radiobiological Protection (ICRP 118), a harmful opacity threshold dose for the eye lens is set at 0.5 Gy [2].

Linear accelerators are the most frequently used equipment in the field of radiation therapy for producing high-energy electrons and photon beams. Electron beam therapy, with a history spanning over half a century, has been widely used in radiation treatment, providing acceptable and consistent radiation doses. It proves particularly effective for treating superficial cancers located within approximately 5 cm of the skin [3].

In electron beam therapy, the electron beam goes through a scattering foil and significantly interacts with various components in the accelerator head and the air between the exit window and the patient, resulting in an undesirably wide penumbra and contaminant photons and scattered electrons which can cause increase dose to depth in tissue [4]. The collimator jaws of the linear accelerator (linac) are inadequate for shaping the electron field since they are positioned at a considerable distance from the patient [4]. To address this issue, electron applicators are commonly used in combination with Cerrobend cutouts. Electron applicators help shaping the electron beam to conform to the contours of the treatment area, typically the tumour [5], and aid in maintaining or improving the beam flatness at the required depth [6]. Applicators serve to confine the electron beam and minimize its lateral dispersion from the accelerator head to the patient’s skin, contributing to the accuracy of treatment delivery [5]. Applicator and cutout options are available in various sizes to accommodate different treatment areas. However, placing anything in an electron pathway, whether air or Cerrobend significantly increases contamination X-rays and scattered electrons. Considering the short mean free path of electrons, scattering occurs both within and outside the applicator, a unique consideration compared to photon-based techniques [7]. Studies examining scattered radiation from different types of electron applicators and various vendors have been documented in the literature [5, 7–23]. Recently, Maharaj et al. [24] conducted a comprehensive systematic literature review on peripheral doses beyond electron applicators in conventional C-arm linear accelerators, comparing different manufacturers such as Varian, Elekta, and Siemens.

However, it is worth noting that a significant portion of these studies are quite dated, with several of them conducted over 20 years ago.

Despite the lower number of cases for electron treatments, considering the progress in electron applicator technology and the introduction of new generations of linear accelerators, there is merit in conducting investigations to explore out-of-field scatter in more contemporary machines. Modern external beam radiotherapy dose determination based on measurements, treatment planning system (TPS), and Monte Carlo (MC) simulations are generally well-established for in-field assessments but challenging for out-of-field regions.

Quantifying this peripheral dose can help with evaluating potential tissue damage. The objective of this study is to examine the peripheral dose associated with an Elekta Versa HD linac and compare the measured data with Monaco TPS. In this study, a novel approach to evaluate peripheral dose is presented. To the authors’ knowledge, to date, there are no reports on comparing the measured peripheral dose and penumbra width with TPS calculations. Cerrosafe, rather than Cerrobend, was employed for the cutouts, and the TPS was adapted using Hogstrom’s pencil-beam electron algorithms. Furthermore, the new electron applicators were specifically engineered to minimize scatter dose [23, 25].

## Materials and methods

Measurements were performed using a clinical Elekta Versa HD machine (Elekta AB, Stockholm, Sweden) operating at 6, 9, and 12 MeV. When the selected electron applicator is connected to the accessory mount within the gantry head, the collimator jaws automatically open to a predefined field size.

### Dosimetry

Gafchromic EBT3 films (Ashland^™^, NJ, USA), were used for dosimetry due to their high-resolution, minimal energy dependence and near-water equivalent radiological properties [26]. To be water equivalent for electron dosimetry, materials should match the linear stopping power and the linear scattering power of water, which is approximately correct if they match the electron density and atomic number of water [27]. Gafchromic EBT3 films have been reported as suitable for measuring megavoltage radiotherapy electron or mixed photon/electron dose distributions in a water phantom, which confirms their water equivalence in these beams. The estimated uncertainty of relative electron dosimetry measurements is reported to be 2.3% (k = 2) for 6 MeV to 16 MeV beams [28].

For film calibration, EBT3 films from the same batch were carefully cut into 4 × 4 cm^2^ pieces using a Beambox-FLUX 40WCO2 laser cutter (Koenig Machinery, VIC, Australia) in a dark room to prevent potential unwanted darkening of the film. Recommendations from the American Association of Physicists in Medicine (AAPM) Task Group 235 report for film handling were followed [29]. Each film piece was affixed to the Plastic Water DT (PWDT: CIRS, Norfolk, VA) phantom using adhesive tape and then irradiated individually with 6 MeV electron beams in a PWDT phantom at a depth of 1.3 cm and at an SSD of 100 cm. Before placing the films, the central beam axis was marked on the Plastic Water DT phantom to ensure accurate alignment. The standard 10 × 10 cm^2^ applicator and cutout were used to expose the films from 1 to 250 monitor units (MUs).

**Fig. 1:**
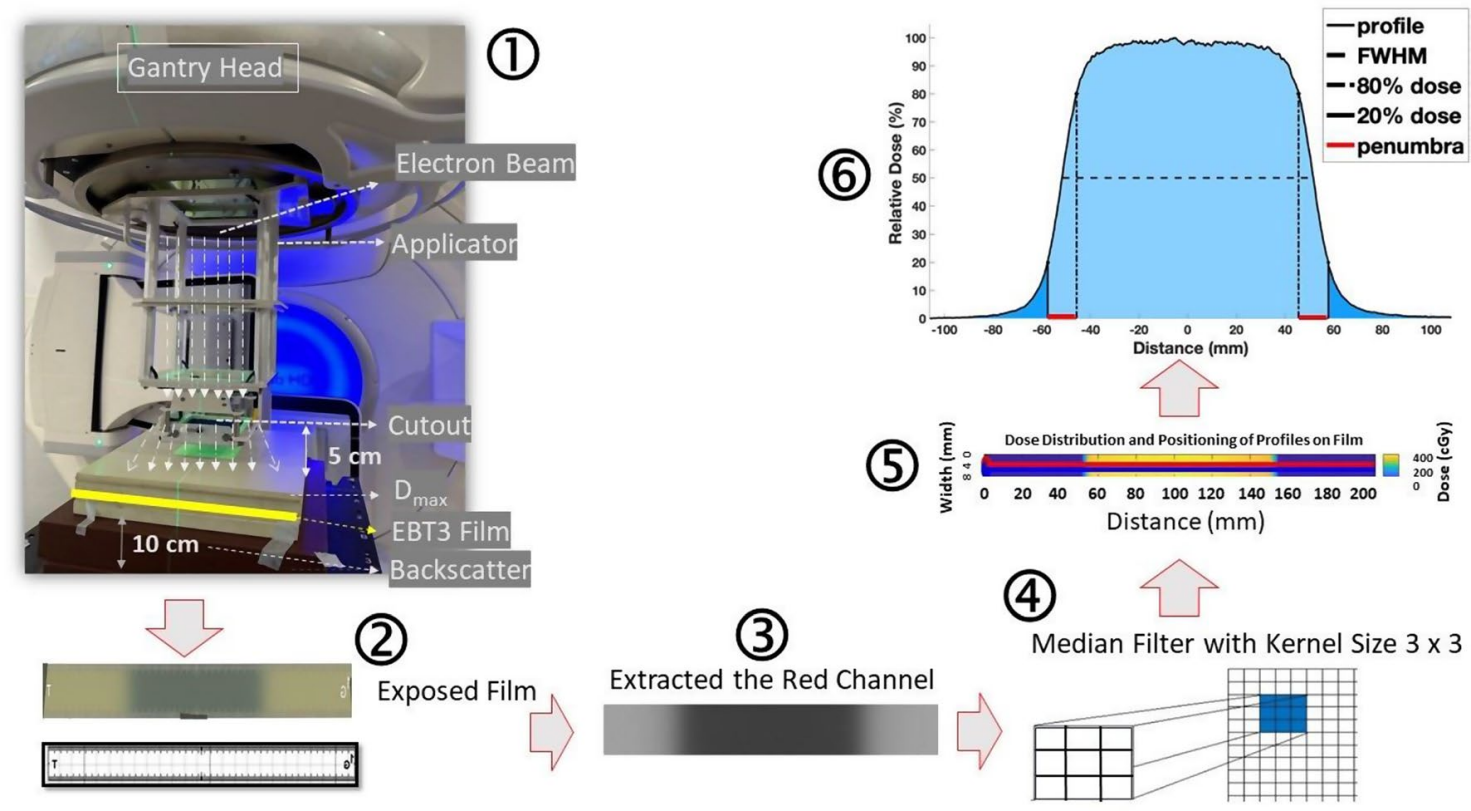
Schematic illustration of the process from measurement to analysis. (1) Measurement setup (note that the vertical dashed lines do not represent the actual particle paths). (2) Exposed film marked and cut using a laser cutter. (3) Film readout. (4) Filter application. (5) Dose profile Extraction. (6) Dose profile with areas defined

Twenty hours after irradiation, each film was scanned using an Epson 12000XL (Nagano, Japan). Each film underwent four scans, and the average of the last three scans was used to account for minor inconsistencies in scanner response. MATLAB software (R2022b, Natick, Massachusetts, USA) was used for image processing and an analytical equation was derived to fit a double exponential curve through results, allowing for the conversion of pixel values from the red channel image (Fig. 1) into dose (cGy). This analytical equation was subsequently used to calculate the out-of-field doses. The measurement setup is shown in Fig. 1.

**Fig. 2:**
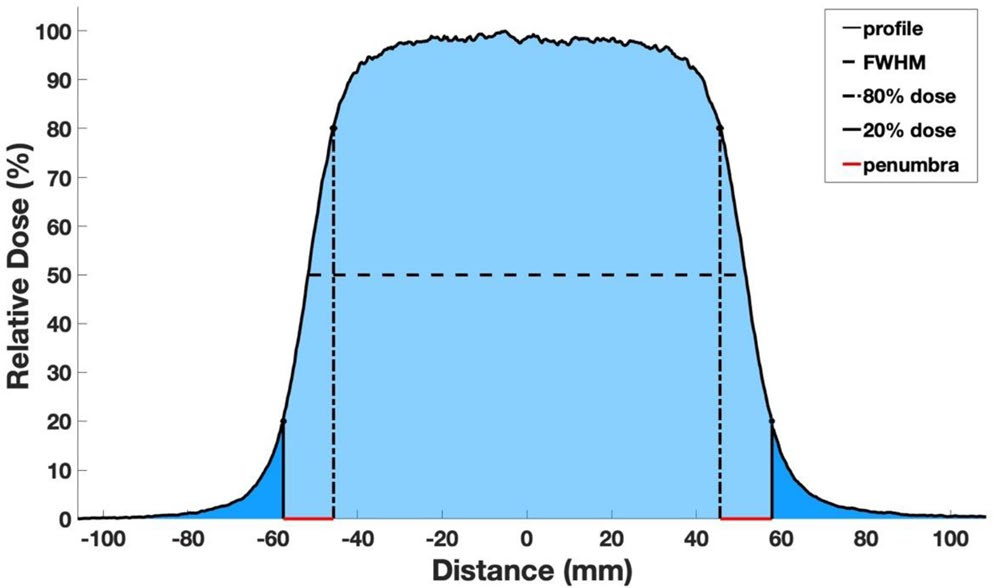
Profile depicting the key parameters, including the Full Width at Half Maximum (FWHM), the peripheral dose (represented by the dark blue area, signifying values falling below 20% of the maximum dose), and the penumbra width (highlighted in red)

**Fig. 3:**
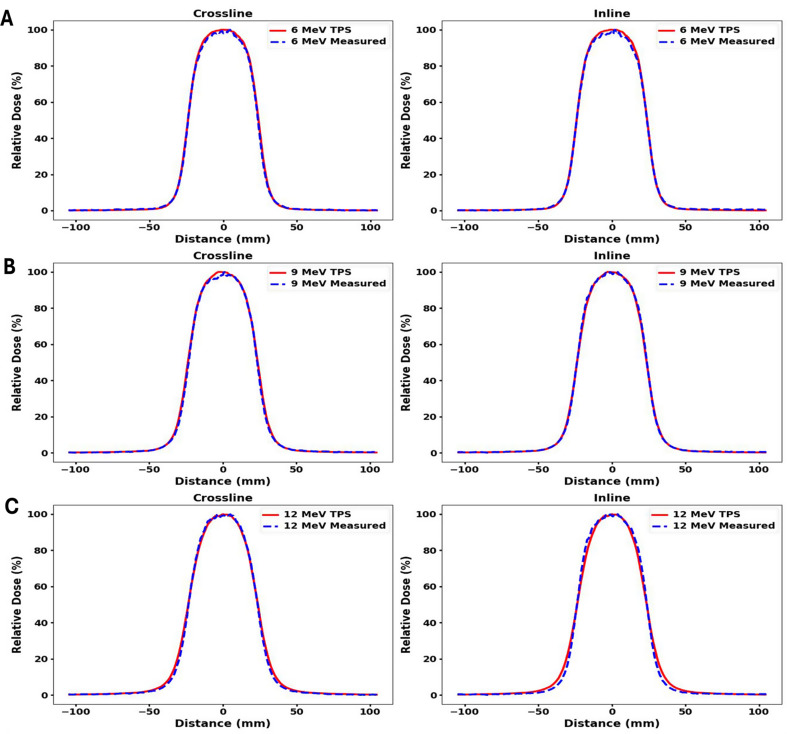
Comparison of in-plane and cross-plane profiles between measured and TPS-calculated data for a 5 cm diameter circle cutout in 10 × 10 cm^2^ applicator. Measurements were made at D_max_ in (**A**) 6 MeV (**B**) 9 MeV and (**C**) 12 MeV beam with gantry and collimator angles set to 0 degrees

### Profile measurements

For the purpose of conducting profile measurements, a ruler-like structure (Fig. 1 ) was created using Fusion 360 software (Autodesk, San Francisco, CA, USA) and used to cut films with a laser cutter. Before placing the film, the central beam axis was marked on the PWDT pahntom to ensure accurate alignment. Each EBT3 film was securely taped to the phantom. Subsequently, the films were exposed to 250 Monitor units (MUs) of 6 MeV, 9 MeV, and 12 MeV beams at the corresponding D_max_ in PWDT phantom: (1.3 cm, 2.1 cm, and 2.9 cm, respectively), at a SSD of 100 cm. The same scanning protocol used for film calibration was followed, measuring profiles up to 110 mm from the central beam axis.

To evaluate the impact of field sizes on peripheral dose and penumbra, exposures were conducted with both the gantry and collimator at 0 degrees, at D_max_ for each energy in plastic water, and the SSD set to 100 cm. 6 × 6 cm^2^, 10 × 10 cm^2^, and 5 cm diameter circular cutouts were used in 10 × 10 cm^2^ applicators. To investigate the effect of collimator angle, measurements were carried out at collimator angles of 0 and 180 degrees with the standard 10 × 10 cm^2^ applicator and cutout. The rest of parameters were similar to the above measurements.

A 10 cm PWDT block (Brown CIRS PW Plastic Water, as shown in Fig. 1) was used to provide backscatter. A 5 cm air gap between the applicator end and the phantom surface was consistently maintained in each setup. Dose profiles were then acquired in both the cross-plane and in-plane directions for each configuration.

Each film was scanned four times, and the last three scans were averaged to ensure the stability of the scanner response. The standard deviation values shown in the graphs represent variations in the scanner response.

In-plane and cross-plane profiles were calculated for the same setups using Monaco^®^ TPS version 6.1.3.0 (Elekta, Stockholm, Sweden). This version of TPS uses MC technique, which is a statistical method based on random numbers, to estimate the dose. The calculation grid spacing was 0.15 cm (isotropic) and the number of histories per cm^2^ was 500,000. The phantom was forced to be treated as water. TPS calculated penumbra width and peripheral doses were compared with measurements.

### Calculation of physical parameters

Before undertaking an evaluation of peripheral doses and penumbra width, beam symmetry was calculated from measured (using films) and TPS-calculated profiles, following the International Electrotechnical Commission (IEC) protocol for electron dosimetry [30]. Based on this protocol, symmetry of a radiation field is defined as the maximum dose ratio at two symmetric points relative to the field’s central axis. Physical penumbra was determined based on the International Commission on Radiation Units and Measurements (ICRU) Report 35 guidelines which defines it as the distance between the 20% and 80% isodose curves (Fig. 2) at the depth of the maximum dose (D_max_) [31]. The mean value of penumbra width on both sides of profiles was calculated for each profile.

1 $$\:\text{S}\text{y}\text{m}\text{m}\text{e}\text{t}\text{r}\text{y}\left(\text{\%}\right)=\:{\left|\frac{\left({\text{D}}_{\left(\text{x},\text{y}\right)}\right)}{{\text{D}}_{\left(-\text{x},-\text{y}\right)}}\right|}_{\text{m}\text{a}\text{x}}\times\:100$$

Where $\:{\left|\frac{\left({D}_{\left(x,y\right)}\right)}{{D}_{\left(-x,-y\right)}}\right|}_{max}$is the maximum ratio from two symmetric points on either side of the central 80% of the full width at half maximum (FWHM) of the profile.

In the context of quantifying peripheral doses during radiation therapy, specific attention was directed towards the region below the 20% isodose threshold. This choice was based on the observation of a sharp fall of the radiation dose as the distance increased from the field edge. This rapid dose fall-off indicates underlying physical processes, potentially involving scattering of electrons and the presence of contaminating photons, although photon contamination mainly affects the dose at deeper depths. Understanding the distribution and magnitude of these sub-threshold peripheral doses is useful, as nearby tissues could be impacted. Collectively, these doses, consisting of scattered electrons and contaminating photons, are commonly referred to as “peripheral doses”. Recognizing the spatial distribution and extent of these peripheral doses is beneficial for the optimization of treatment plans and the minimization of unintended radiation exposure to non-targeted anatomical structures. In this study, peripheral dose was calculated using Eq. (2):

2 $$\:\text{P}\text{e}\text{r}\text{i}\text{p}\text{h}\text{e}\text{r}\text{a}\text{l}\:\text{D}\text{o}\text{s}\text{e}\:\left(\text{\%}\right)=\:\left(\frac{\text{D}\text{a}\text{r}\text{k}\:\text{B}\text{l}\text{u}\text{e}\:\text{A}\text{r}\text{e}\text{a}}{\text{C}\text{e}\text{n}\text{t}\text{r}\text{a}\text{l}\:\text{L}\text{i}\text{g}\text{h}\text{t}\:\text{B}\text{l}\text{u}\text{e}\:\text{A}\text{r}\text{e}\text{a}}\right)$$

## Results

### Profiles

**Fig. 4:**
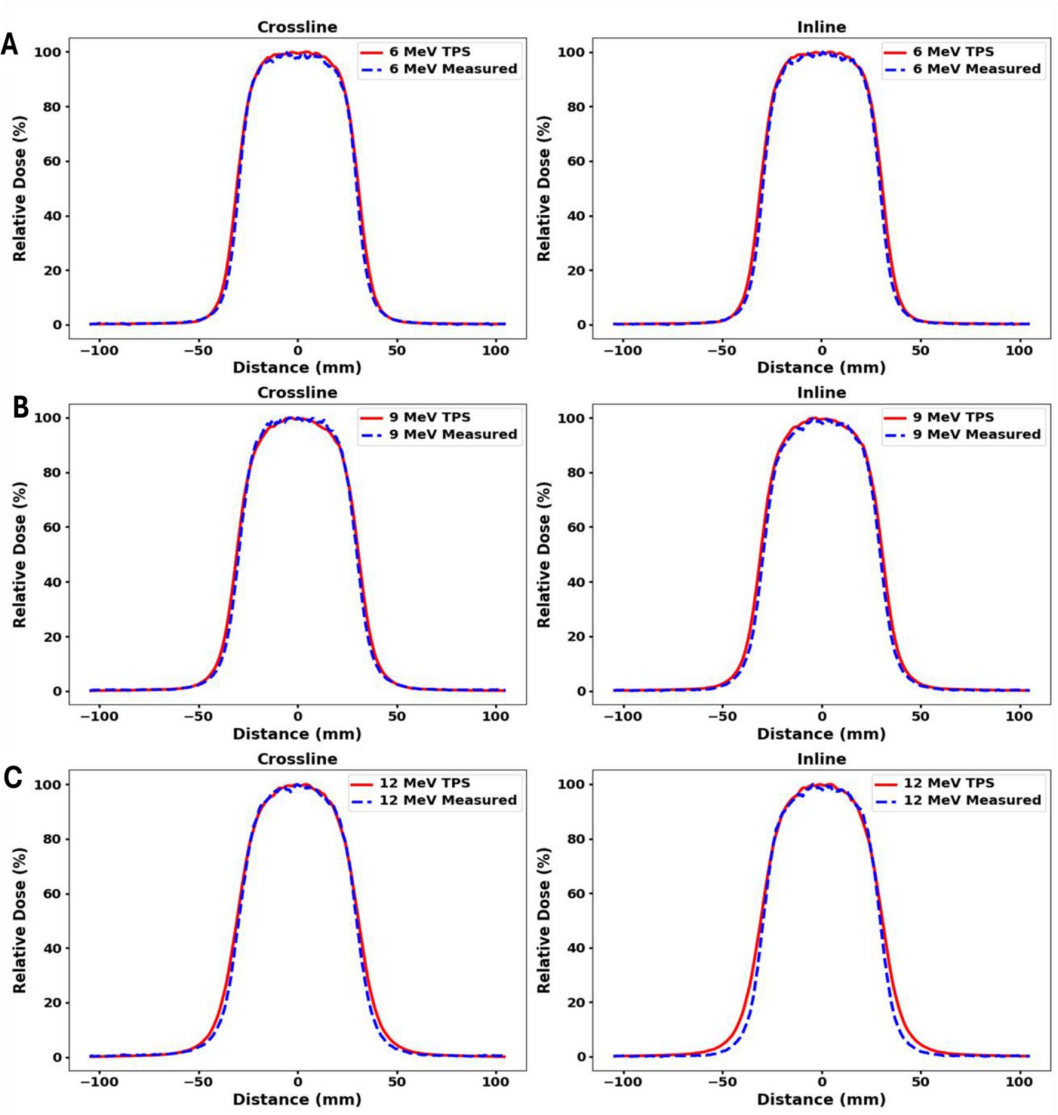
Comparison of in-plane and cross-plane profiles between measured and TPS-calculated data for a 6 × 6 cm^2^ cutout in 10 × 10 cm^2^ applicator. Measurements were made at D_max_ in (**A**) 6 MeV (**B**) 9 MeV and (**C**) 12 MeV beam with gantry and collimator angles set to 0 degree

**Fig. 5:**
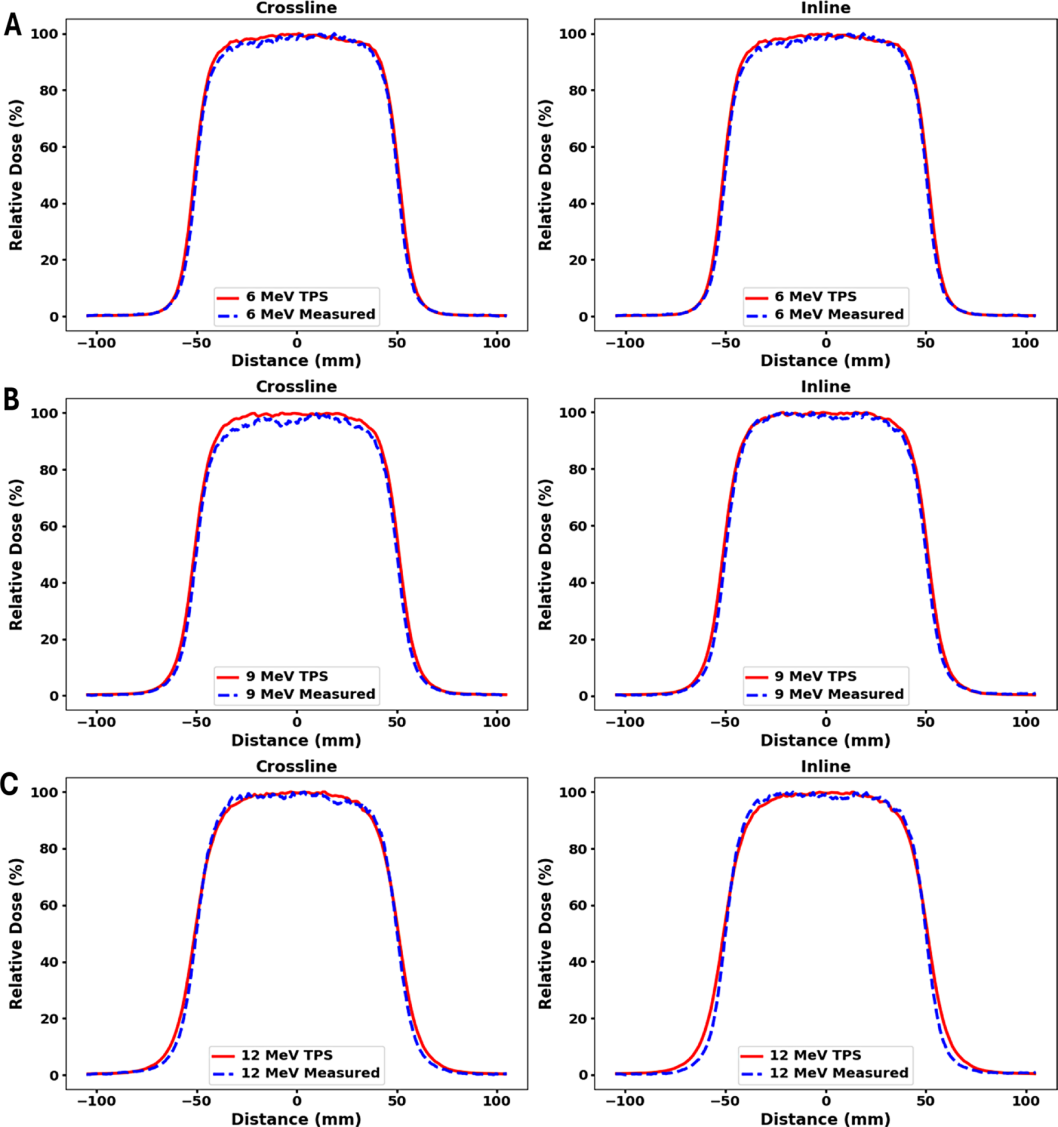
Comparison of in-plane and cross-plane profiles between measured and TPS-calculated data for a 10 × 10 cm^2^ cutout in 10 × 10 cm^2^ applicator. Measurements were made at D_max_ in (**A**) 6 MeV (**B**) 9 MeV and (**C**) 12 MeV beam with gantry and collimator angles set to 0 degree

**Fig. 6:**
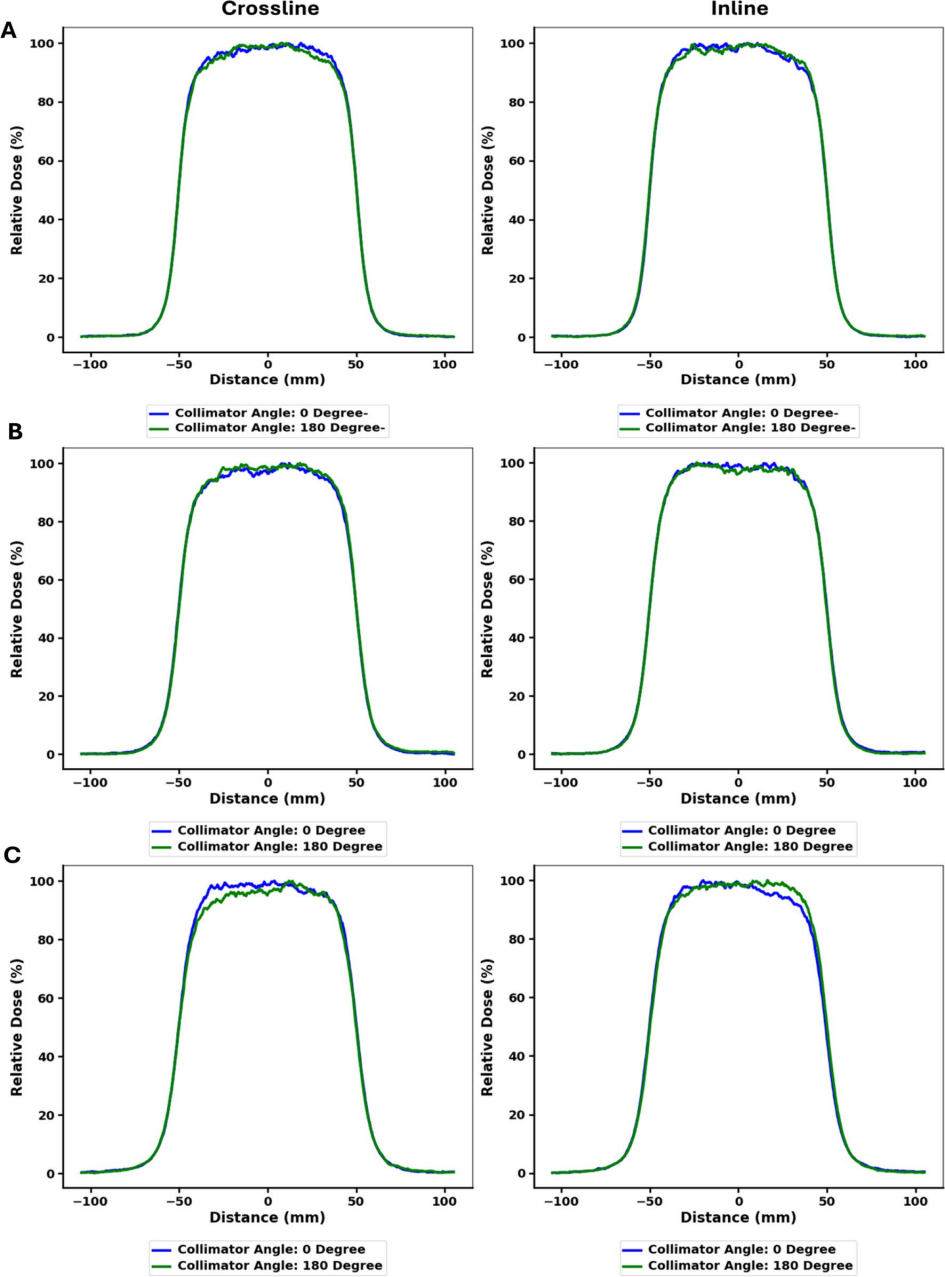
In-plane and cross-plane profiles for 0- and 180-degrees collimator angles. Measurements were performed at D_max_ in a (**A**) 6 MeV (**B**) 9 MeV and (**C**) 12 MeV beam with 10 × 10 cm² cutout and applicator and gantry angle set to 0 degree

Measured and TPS calculated profiles for 5 cm circle, 6 × 6 cm^2^, and 10 × 10 cm^2^ cutouts within a 10 × 10 cm^2^ applicator for 6 MeV, 9 MeV and 12 MeV are shown in Figs. 3 and 4, and Fig. 5 respectively. Both inline and crossline profiles were measured. At 12 MeV, TPS profiles exhibit outward bulging compared to the measured profiles. Figure 6 shows measured profiles for 0 and 180 degree collimator angles for all three energies.

### Symmetry

Symmetry was calculated using Eq. (1). Table 1 shows the symmetry values for both measured and TPS-calculated profiles with field sizes of a 5 cm diameter circle, 6 × 6 cm², and 10 × 10 cm². These measurements were taken with an applicator size of 10 × 10 cm² at D_max_ of 6, 9, and 12 MeV. Table 2 shows the symmetry values for the measured profiles at 0° and 180° collimator angles, using the same applicator and field size of 10 × 10 cm² at D_max_ of 6, 9, and 12 MeV. The symmetry of the measured profiles was found to be within ±3%, meeting the acceptable standards outlined by the IEC electron dosimetry protocol [30].

**Table 1:** Symmetry values for measured and TPS-calculated profiles at D_max_ of each energy, with a consistent applicator size 10 × 10 cm^2^, and with the gantry and collimator angle set to 0 degree

Energy (MeV)	Crossline/Inline	Cut-out Size (cm^2^)	Measured Symmetry (Average$\:\pm\:$SD)	TPS Symmetry
6	Crossline	10 × 10	100.2$\:\pm\:$ 0.2	101.1
6	Inline	10 × 10	101.8$\:\pm\:$ 0.5	100.8
6	Crossline	6 × 6	102.1$\:\pm\:$ 0.2	100.8
6	Inline	6 × 6	101.5$\:\pm\:$ 0.7	100.8
6	Crossline	Circle (d = 5 cm)	101.16$\:\pm\:$ 0.60	101.4
6	Inline	Circle (d = 5 cm)	101.6$\:\pm\:$ 0.4	100.8
9	Crossline	10 × 10	100.4 $\:\pm\:$0.1	100.8
9	Inline	10 × 10	101.8 $\:\pm\:$0.9	100.7
9	Crossline	6 × 6	101.9 $\:\pm\:$0.7	100.8
9	Inline	6 × 6	101.4 $\:\pm\:$0.3	100.9
9	Crossline	Circle (d = 5 cm)	101.9 $\:\pm\:$1.1	101.3
9	Inline	Circle (d = 5 cm)	101.9$\:\pm\:$ 0.6	101.9
12	Crossline	10 × 10	101.5 $\:\pm\:$0.5	100.8
12	Inline	10 × 10	101.9 $\:\pm\:$0.6	101.5
12	Crossline	6 × 6	101.3 $\:\pm\:$0.2	100.7
12	Inline	6 × 6	102.2$\:\pm\:$ 0.4	100.8
12	Crossline	Circle (d = 5 cm)	101.8$\:\pm\:$ 0.2	101.2
12	Inline	Circle (d = 5 cm)	102.7$\:\pm\:$ 0.1	100.2

**Table 2:** Symmetry measured at D_max_ of each energy, with a consistent applicator and cutout size of 10 × 10 cm^2^, and with the gantry angle set to 0 degree

Energy (MeV)	Crossline/Inline	Collimator Angle (Degree)	Measured Symmetry (Average$\:\pm\:$SD)
6	Crossline	0	100.2$\:\pm\:$0.2
6	Inline	0	101.8$\:\pm\:$0.5
6	Crossline	180	101.8$\:\pm\:$0.2
6	Inline	180	103.2$\:\pm\:$0.5
9	Crossline	0	100.4$\:\pm\:$0.1
9	Inline	0	101.8$\:\pm\:$0.9
9	Crossline	180	101.4$\:\pm\:$0.1
9	Inline	180	103.1$\:\pm\:$0.3
12	Crossline	0	101.5$\:\pm\:$0.5
12	Inline	0	101.9$\:\pm\:$0.6
12	Crossline	180	100.0$\:\pm\:$0.1
12	Inline	180	101.0$\:\pm\:$0.2

### Effect of field sizes on penumbra width and peripheral doses

**Fig. 7:**
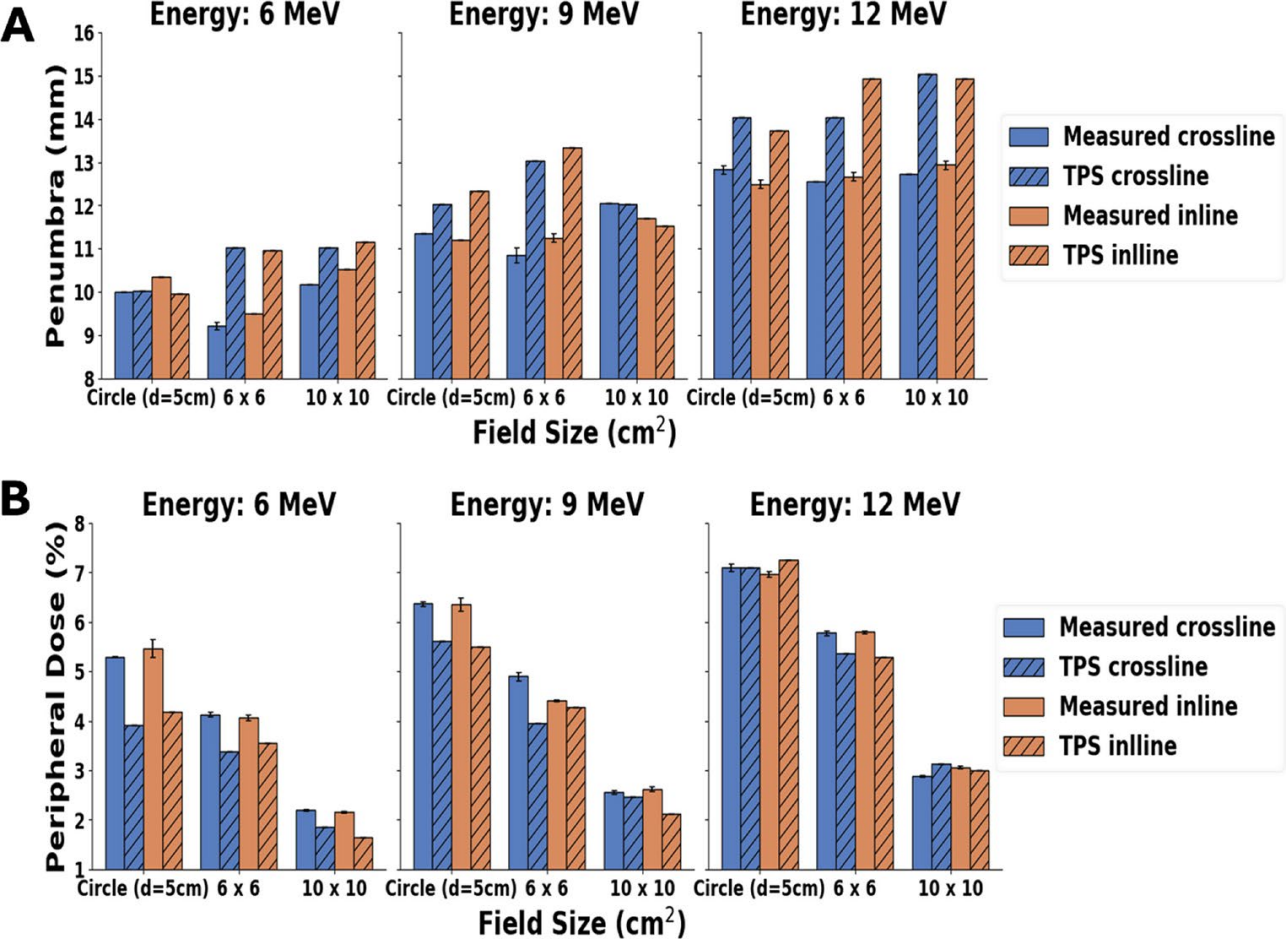
Comparative analysis between the measured and TPS-calculations for (**A**) the physical penumbra widths and (**B**) peripheral doses at the depth of maximum dose (D_max_). The evaluations were performed for three different cutouts (Circle 5 cm diameter, 6 × 6 cm², and 10 × 10 cm²) ina 10 × 10 cm² applicator. Assessments were carried out in 6 MeV, 9 MeV, and 12 MeV beams, with both gantry and collimator angles set to 0 degrees

**Fig. 8:**
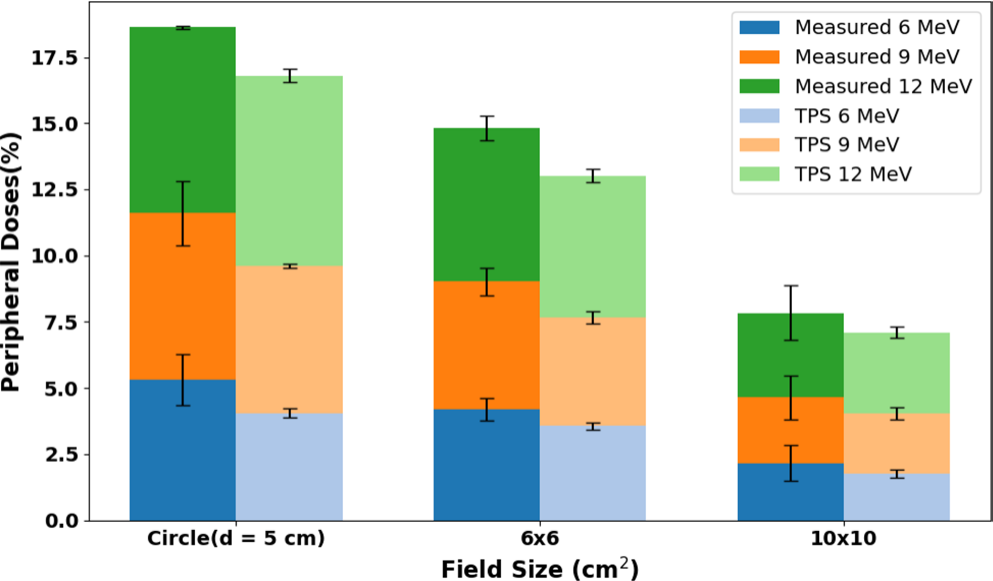
Peripheral doses for all field sizes across all energies for measured and TPS-calculated profiles. Peripheral dose values are averaged for crossline and inline profiles. Vertical lines indicate the standard deviation between crossline and inline peripheral dose values

The influence of field size on penumbra width and peripheral doses across various energy levels is shown in Fig. 7, which compares the measured and TPS-calculated profiles for the 6 × 6, and 10 × 10 cm^2^ cutouts, and the 5 cm diameter circular cutout. Furthermore, Fig. 8 shows the comparison of averaged inline and crossline peripheral doses for all field sizes across all energies, comparing measured and calculated profiles. As the energy increases, there is an expected increase in peripheral dose values for the measured profiles.

### Effect of collimator angle on penumbra width and peripheral dose

**Fig. 9:**
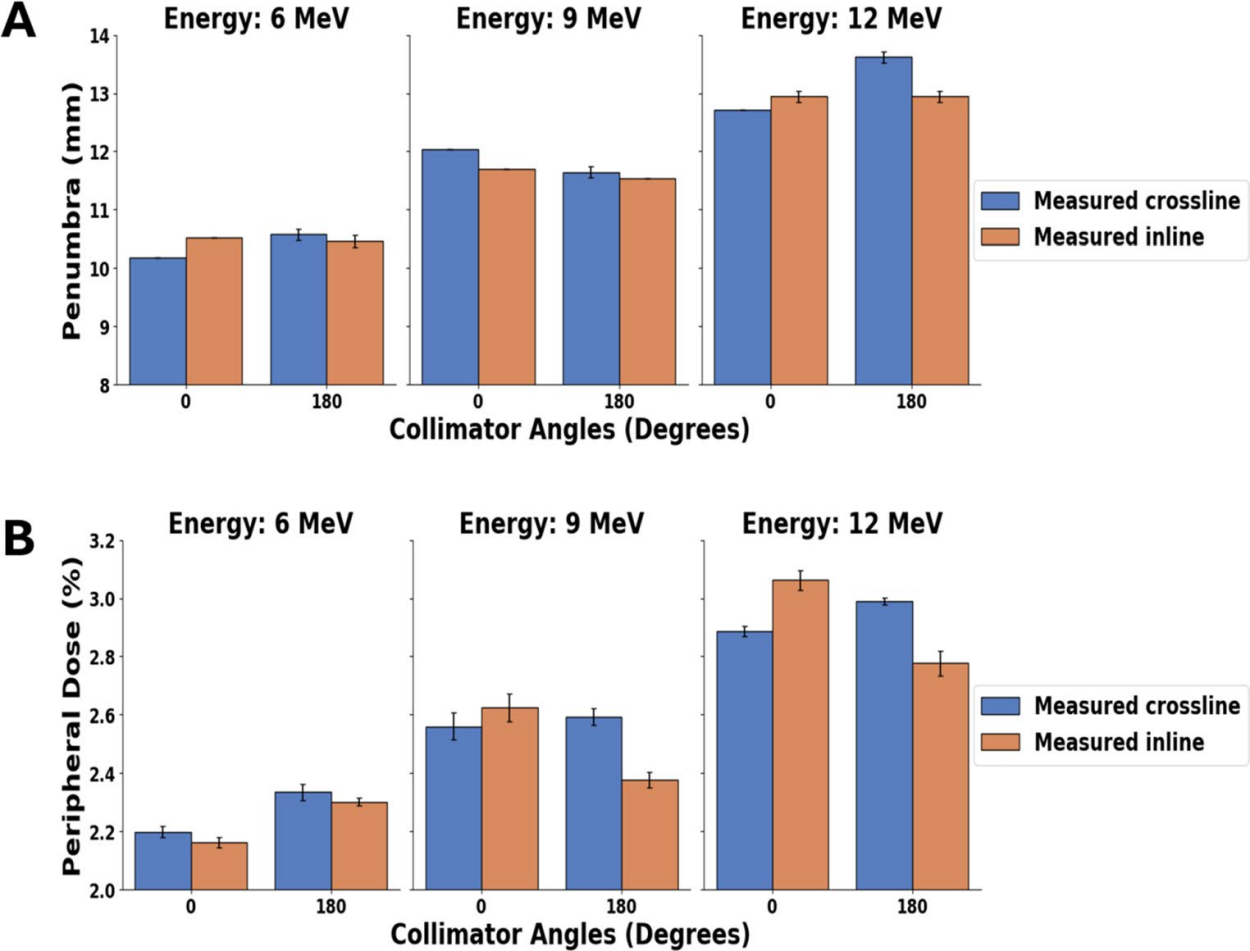
Measured (**A**) Penumbra width (**B**) Peripheral dose at D_max_ using 0 and 180-degree collimator angles for a 10 × 10 cm^2^ applicator and cutout for 6, 9 and 12 MeV beams at gantry 0 degree

The effect of the collimator angle on penumbra width and peripheral dose is shown in Fig. 9 for consistent field and applicator sizes across all tested energy levels.Penumbra width and peripheral doses were calculated from inline and crossline profiles, which were measured at D_max_ with collimator angles of 0 and 180 degrees for a 10 × 10 cm^2^ applicator and cutout for 6, 9 and 12 MeV beams at gantry 0 degree..

### Peripheral dose fall off with distance from applicator edge

**Fig. 10:**
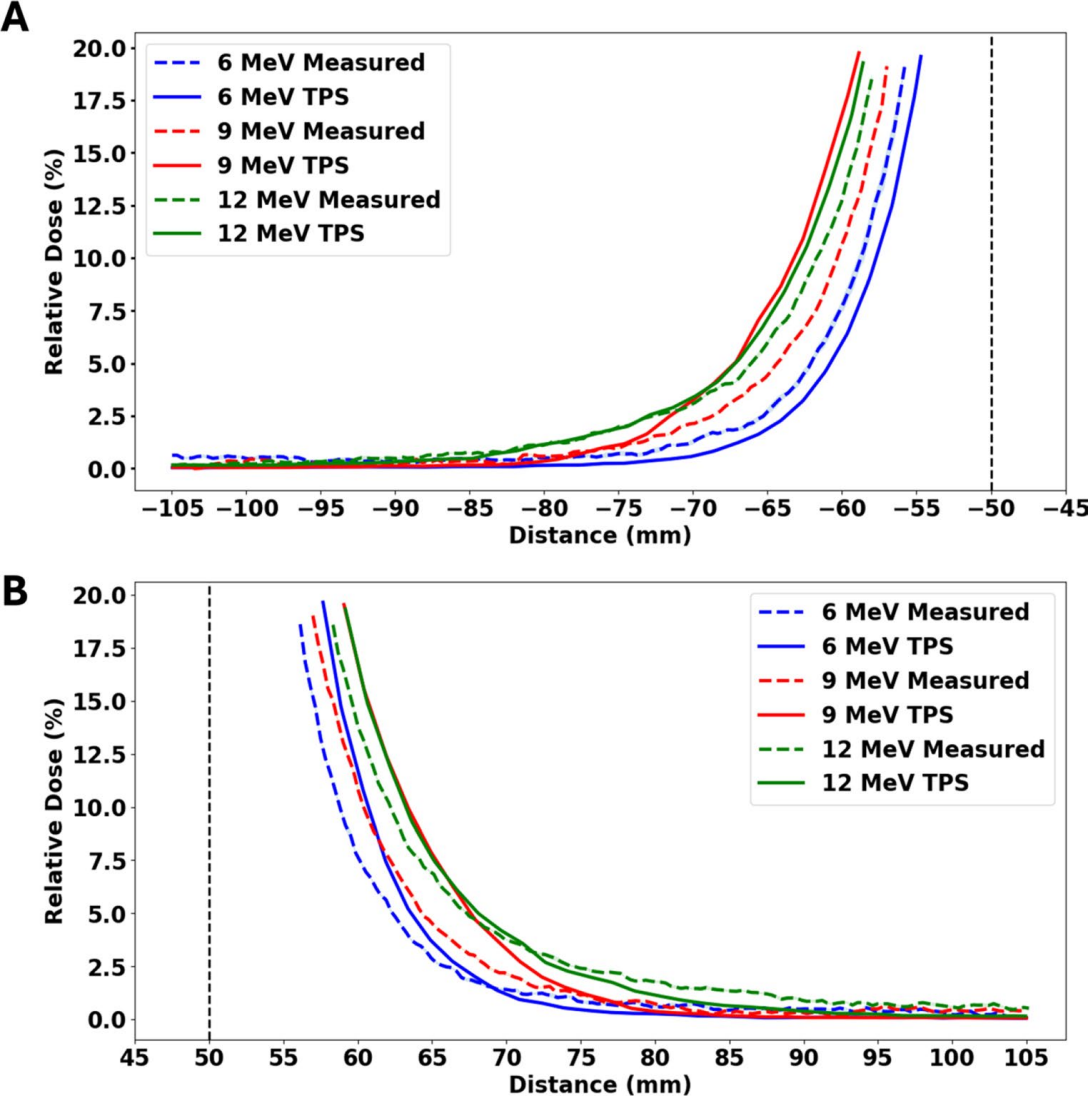
The crossline out of field profiles measured and calculated using Monaco TPS in 6, 9 and 12 MeV electron beams, using a standard 10 × 10 cm^2^ applicator and cutout, with gantry and collimator angles set to 0 degree measured at D_max_ for each beam. (**A**) left side (**B**) right side. Note: Vertical lines indicate the field edge

Figure 10 shows the comparison between measured and TPS-calculated dose profiles below 20% for 6, 9, and 12 MeV electron beams at varying distances from the applicator edge. The out-of-field dose decreases with increasing distance from the applicator edge, as expected. Between 10 and 15 mm from the applicator edge, the out-of-field doses were less than 5% of the maximum dose.

## Discussion

Peripheral dose may result from scattered photon or electron components. Bremsstrahlung photons are generated upon the interaction between a high-energy electron beam and parts of the applicator. The electron component contributes to the peripheral dose through three primary mechanisms: (1) scattering out of the applicator, (2) penetration through collimating structures within the applicator, and (3) direct exit into the surrounding air without interacting with applicator components [7]. Notably, the latter two mechanisms—penetration and direct escape—are more common with higher-energy beams, while scattering is characteristic of lower-energy beams which includes scattered electrons and bremsstrahlung photons [8, 32]. The energy spectrum of scattered photons and electrons depends on the field size, distance from the applicator, and material of the applicator, which can be approximated using MC simulations [7, 33]. The main source of the peripheral dose is known to be the photons produced in the Bremsstrahlung effect [13].

### Symmetry

The symmetry of the measured was found within the range of ±3% aligning with the acceptable standards stipulated by the IEC protocol [30]. This investigation confirmed the uniform delivery of the dose across the treatment field. It was essential to ensure that the radiation hit the center of the film, as it contributed to the symmetrical profile and facilitated the calculation of the peripheral dose.

### Effect of field sizes on penumbra width and peripheral doses

#### Penumbra width

Figure 7 (A) indicated that the penumbra value showed an increasing trend with energy. Specifically, for a field size measured in crossline and inline at 6 MeV, the average penumbra width was 10.0 ± 0.5 mm, while at 12 MeV, it was 12.7 ± 0.1 mm. Interestingly, TPS-predicted values were slightly higher than measured. The root mean square deviation (RMSD) of the measured and TPS-calculated values across all fields and energies for both crossline and inline, was 1.8 mm on an average.

#### Peripheral dose

Figure 7 (B), clearly shows that on average, the peripheral dose values decrease with increasing field size. These results are consistent with the report by Cardenas et al. [34]. The peripheral dose values for the TPS and measurements were found to be close to each other, except for the circular cutout in 6 MeV, where differences greater than 1% were observed. On average, the RMSD value between the measured and TPS values is 1.1%, indicating no significant discrepancy in the peripheral dose values between the two, as Figs. 3 and 4, and Fig. 5 shows through the comparison of in-plane and cross-plane profiles for all three field sizes and for all three energies.

### Effect of collimator angles on penumbra width and peripheral doses

#### Penumbra width

**Fig. 11:**
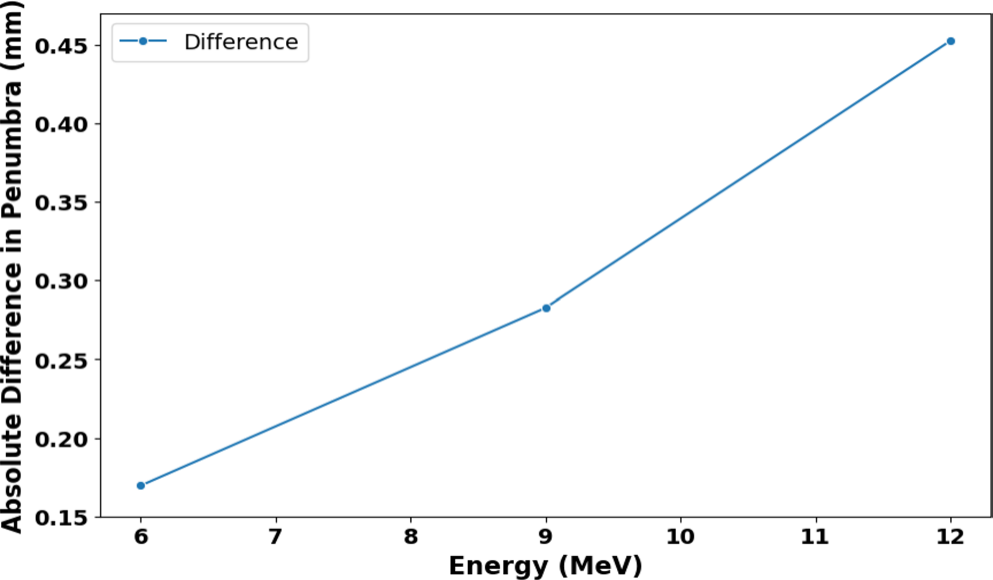
The absolute difference in penumbra width between the two collimator angles (0 and 180 degrees) for the average of crossline and inline measurements for 6, 9 and 12 MeV

In Fig. 9 (A), the average penumbra value for collimator angles 0 and 180 degrees is 10.4 ± 0.2 mm in 6 MeV, 11.7 ± 0.2 mm in 9 MeV, and 13.1 ± 0.4 mm in 12 MeV. The penumbra width increased with energy. In both crossline and inline, the penumbra width for each collimator angle remained notably consistent in 6 and 9 MeV beams. However, at 12 MeV, the 180-degree angle exhibited a slight difference, which could be due to the accelerated beam not perfectly coinciding with the collimator axis. Comparing the average differences in inline and crossline penumbra widths across both collimator angles, there is an increasing trend in differences (0.17 mm, 0.28 mm and 0.45 mm for 6 MeV, 9 MeV and 12 MeV respectively) with increasing energy, as shown in Fig. 11, which shows the absolute difference in penumbra width for each energy’s collimator angles for the averaged crossline and inline measurements. Figure 11, showing a noticeable trend despite relatively small numerical variations (~ 0.3 mm). These differences can be attributed to the beam focal spot not aligning perfectly with the collimator centre of rotation. This effect is anticipated to be more pronounced with higher energy beams, as discussed by Riis et al. [35].

#### Peripheral dose

Figure 9 (B), illustrates that the peripheral percentage dose remains nearly identical for the 0 and 180-degree collimator across all energies, indicating consistent peripheral doses for each energy between the 0 and 180-degree collimator angles, which can be seen in Fig. 6 which shows the in-plane and cross-plane profiles for 0 and 180 degree collimator angle for 6 MeV electron beam at 10 × 10 cm^2^ field size. 

### Peripheral dose fall off with distance from applicator edge

Overall, in the out-of-field region, doses were higher near the field edge, as expected. With increasing beam energy, the curves bulged outward, which means higher peripheral doses. It was noted that from 10 to 15 mm from the field edge up to about 110 mm, peripheral dose values were less than 5% of maximum dose (Fig. 10). These results are in agreement with the findings of Cardenas et al. [34] and Haghparast et al. [16]. The clinical significance of the dose being less than 5% of the maximum dose varies with the lesion location. For instance, areas near sensitive structures such as the eye may pose greater clinical concern compared to lesions located on less critical areas like the arm.

In certain electron treatments, sensitive organs like the eyes and thyroid may lie near the treatment field edge, making it important to evaluate the out-of-field dose. ICRP report 118specifies a threshold dose of 0.5 Gy for the eye lens [2]. The threshold dose of 0.5 Gy is likely to be reached clinically in areas close to the edge of the treatment field in high-energy electron beams, due to increased scatter and penetration. Larger treatment fields and the use of multiple fields or varying beam angles can cumulatively increase the dose to surrounding tissues. Surface doses can be higher due to electron scatter from the patient’s body or treatment couch, potentially leading to out-of-field areas receiving doses around 0.5 Gy. Electron beams have limited penetration compared to photons, potentially resulting in increased dose to skin. Skin, a sensitive organ [36], is consistently affected during electron treatments, underscoring the significance of out-field doses.

While out-of-field electron doses may seem less significant than photon beams, they should be considered in electron therapy due to the absence of a secondary cancer threshold, with dose values dependent on energy, applicator dimensions, and depth [22].

## Conclusion

This study investigated the dependence of the peripheral dose and penumbra width of electron beams on the cutout size, and collimator angle. On average, both the penumbra width and peripheral dose increased with beam energy across all field sizes and collimator angles. For each energy, peripheral doses and penumbra widths did not exhibit differences across varying collimator angles. The TPS showed higher values for penumbra width in comparison to the measured values. However, RMSD of 1.8 was calculated for the measured and TPS-calculated peripheral doses. Approximately 10 to 15 mm away from the field edge, peripheral doses reduced to less than 5% of the maximum dose, suggesting that they could be easily removed by implementing additional shielding for patients [37], where needed. Understanding peripheral dose values can assist the radiotherapy team in safeguarding critical organs outside the applicator.

## Data Availability

Not applicable.

## Abbreviations

CAX: Central Axis
D_max_: Depth of Maximum Dose
SEER: Epidemiology and End Results
FWHM: Full Width at Half Maximum
IEC: International Electrotechnical Commission
ICRU: International Commission on Radiation Units and Measurements
ICRP: International Committee for Radiobiological Protection
LINAC: Linear Accelerator
MC: Monte Carlo
MUs: Monitor Units
RMSD: Root Mean Square Deviation
SSD: Source-to-Surface Distance
PWDT: Plastic Water DT Phantom
TPS: Treatment Planning System

## References

[1] Dracham CB, Shankar A, Madan R (2018) Radiation induced secondary malignancies: a review Article. Radiat Oncol J 36(2):85–94 29983028 10.3857/roj.2018.00290 PMC6074073

[2] ICRP, Stewart FA, Akleyev AV et al (2012) ICRP publication 118: ICRP statement on tissue reactions and early and late effects of radiation in normal tissues and organs–threshold doses for tissue reactions in a radiation protection context. Ann ICRP 41:1–322 10.1016/j.icrp.2012.02.001 22925378

[3] Khan FM, Gibbons JP (2014) Khan’s the physics of radiation therapy. 5th Edition ed. Philadelphia: Lippincott Williams & Wilkins

[4] Podgoršak EB (2006) Radiation physics for medical physicists. Biological and medical physics, biomedical engineering. Springer, Berlin;

[5] Yeboah C et al (2010) Quantification and reduction of peripheral dose from leakage radiation on Siemens primus accelerators in electron therapy mode. J Appl Clin Med Phys 11(3):3105 20717080 10.1120/jacmp.v11i3.3105 PMC5720440

[6] Dragun AE, Brady LW, Yaeger TE et al (2013) Springer Berlin Heidelberg: Berlin, Heidelberg. 198–206

[7] Chow JC, Grigorov GN (2006) Peripheral dose outside applicators in electron beams. Phys Med Biol 51(12):N231–N240 16757855 10.1088/0031-9155/51/12/N01

[8] Shimozato T et al (2013) Monte Carlo simulation and measurement of radiation leakage from applicators used in external electron radiotherapy. Phys Med 29(4):388–396 22771332 10.1016/j.ejmp.2012.06.006

[9] Lin JP et al (2001) The measurement of photoneutrons in the vicinity of a Siemens primus linear accelerator. Appl Radiat Isot 55(3):315–321 11515653 10.1016/s0969-8043(01)00084-7

[10] Biltekin F, Yeginer M, Ozyigit G (2015) Investigating in-field and out-of-field neutron contamination in high-energy medical linear accelerators based on the treatment factors of field size, depth, beam modifiers, and beam type. Phys Med 31(5):517–523 25873196 10.1016/j.ejmp.2015.03.015

[11] Keys RA, Purdy JA (1984) Radiation leakage from Linac electron applicator assembly. Int J Radiat Oncol Biol Phys 10(5):713–721 6429095 10.1016/0360-3016(84)90302-x

[12] Pennington EC, Jani SK, Wen BC (1988) Leakage radiation from electron applicators on a medical accelerator. Med Phys 15(5):763–765 3141759 10.1118/1.596191

[13] Iktueren B et al (2012) The peripheral dose outside the applicator in electron beams of Oncor linear accelerator. Radiat Prot Dosimetry 150(2):192–197 22025738 10.1093/rpd/ncr392

[14] Karbaf M et al (2019) Assessment of radiation leakage from treatment applicator of Siemens primus plus and Siemens artiste linear accelerators. J Cancer Res Ther 15(1):216–222 30880781 10.4103/jcrt.JCRT_1096_16

[15] Perec A, Kubo H (1990) Radiation leakage through electron applicators on Clinac-1800 accelerators. Med Phys 17(4):715–719 2215419 10.1118/1.596472

[16] Haghparast A et al (2018) The peripheral dose outside the applicator in electron beams of an Elekta linear accelerator. Australas Phys Eng Sci Med 41(3):647–655 29943310 10.1007/s13246-018-0660-9

[17] Alabdoaburas MM et al (2015) Experimental assessment of out-of-field dose components in high energy electron beams used in external beam radiotherapy. J Appl Clin Med Phys 16(6):435–448 26699572 10.1120/jacmp.v16i6.5616 PMC5691002

[18] Schneider AJ (1982) Radiation leakage from electron applicator assembly on a linear accelerator. Med Phys 9(5):761–762 7155080 10.1118/1.595125

[19] van der Laarse R, Bruinvis IA, Nooman MF (1978) Wall-scattering effects in electron beam collimation. Acta Radiol Oncol Radiat Phys Biol 17(2):113–124 99983 10.3109/02841867809127912

[20] van Battum LJ, van der Zee W, Huizenga H (2003) Scattered radiation from applicators in clinical electron beams. Phys Med Biol 48(15):2493–2507 12953911 10.1088/0031-9155/48/15/316

[21] Cardenas CE et al (2016) Out-of‐field doses and neutron dose equivalents for electron beams from modern varian and Elekta linear accelerators. J Appl Clin Med Phys 17(4):442–455 27455499 10.1120/jacmp.v17i4.6216 PMC5690067

[22] Gul OV (2024) Experimental evaluation of out-of-field dose for different high-energy electron beams and applicators used in external beam radiotherapy. Radiat Phys Chem 215:111345

[23] Sengupta B et al (2024) Out of field scatter from electron applicator in modern linear accelerators. J Appl Clin Med Phys 24(6):e14265 10.1002/acm2.14265 PMC11163494 38335230

[24] Maharaj KD et al (2024) Peripheral doses beyond Electron applicators in conventional C-Arm linear accelerators: A systematic literature review. Technol Cancer Res Treat 23:15330338241239144 38515394 10.1177/15330338241239144 PMC10958816

[25] Narayanasamy G et al (2016) Commissioning an Elekta versa HD linear accelerator. J Appl Clin Med Phys 17(1):179–191 26894351 10.1120/jacmp.v17i1.5799 PMC5690217

[26] Santos T, Ventura T, Lopes MD (2021) A review on radiochromic film dosimetry for dose verification in high energy photon beams. Radiat Phys Chem 179:109217

[27] Podgorsak EB (2005) Radiation oncology physics. IAEA Vienna

[28] Sipila P et al (2016) Gafchromic EBT3 film dosimetry in electron beams - energy dependence and improved film read-out. J Appl Clin Med Phys 17(1):360–373 26894368 10.1120/jacmp.v17i1.5970 PMC5690204

[29] Niroomand-Rad A et al (2020) Report of AAPM task group 235 radiochromic film dosimetry: an update to TG-55. Med Phys 47(12):5986–6025 32990328 10.1002/mp.14497

[30] IEC, Medical Electron Accelerators-Functional performance characteristics. (2007) IEC Geneva, Switzerland

[31] Dosimetry R (1984) Electron beams with energies between 1 and 50 mev. ICRU Rep 35:14–15

[32] Soto-Bernal TG et al (2017) Neutron production during the interaction of monoenergetic electrons with a tungsten foil in the radiotherapeutic energy range. Nuclear instruments & methods in physics research section a-Accelerators spectrometers detectors and associated equipment. 868:27–38

[33] Douk HS et al (2018) Accuracy evaluation of distance inverse square law in determining virtual electron source location in Siemens primus Linac. Rep Pract Oncol Radiother 23(2):105–113 29681773 10.1016/j.rpor.2018.01.002 PMC5908270

[34] Cardenas CE et al (2016) Out-of-field doses and neutron dose equivalents for electron beams from modern varian and Elekta linear accelerators. J Appl Clin Med Phys 17(4):442–455 27455499 10.1120/jacmp.v17i4.6216 PMC5690067

[35] Riis HL et al (2024) Assessing focal spot alignment in clinical linear accelerators: a comprehensive evaluation with triplet phantoms. Phys Eng Sci Med 47:1–23 10.1007/s13246-024-01450-9 PMC11666691 38954381

[36] Soriano JL et al (2019) Therapy for prevention and treatment of skin ionizing radiation damage: a review. Int J Radiat Biol 95(5):537–553 10.1080/09553002.2019.1562254 30570420

[37] He H et al (2021) Shielding effect of a lead apron on the peripheral radiation dose outside the applicator of electron beams from an Elekta linear accelerator. J Appl Clin Med Phys 22(1):327–336 10.1002/acm2.13089 PMC7856487 33296548

